# Detecting latent topics and trends in blended learning using LDA topic modeling

**DOI:** 10.1007/s10639-022-11118-0

**Published:** 2022-06-06

**Authors:** Bin Yin, Chih-Hung Yuan

**Affiliations:** School of Economics and Commerce, University of Electronic Science and Technology of China, Zhongshan Institute, Zhongshan, China

**Keywords:** Blended learning, Flipped classroom, Hybrid course, LDA topic modeling, Topic trends, Word cloud

## Abstract

With the rapid application of blended learning around the world, a large amount of literature has been accumulated. The analysis of the main research topics and development trends based on a large amount of literature is of great significance. To address this issue, this paper collected abstracts from 3772 eligible papers published between 2003 and 2021 from the Web of Science core collection. Through LDA topic modeling, abstract text content was analyzed, then 7 well-defined research topics were obtained. According to the topic development trends analysis results, the emphasis of topic research shifted from the initial courses about health, medicine, nursing, chemistry and mathematics to learning key elements such as learning outcomes, teacher factors, and presences. Among 7 research topics, the popularity of presences increased significantly, while formative assessment was a rare topic requiring careful intervention. The other five topics had no significant increase or decrease trends, but still accounted for a considerable proportion. Through word cloud analysis technology, the keyword characteristics of each stage and research focus changes of research were obtained. This study provides useful insights and implications for blended learning related research.

## Introduction

By combining the advantages of traditional face-to-face teaching and emerging online learning, blended learning has become more widely used (Liu et al., 2016). Especially during the COVID-19 pandemic, the application of blended learning has grown rapidly worldwide (Ashraf et al., 2021). There have been plenty of related studies on blended learning from different perspectives. Due to the accumulation of a large number of relevant literature, it is necessary to sort out the research topics. In addition, the exploration of the evolution history of the topic helps to further predict the topic development trends.

Blended learning is defined as “a thoughtful integration of classroom face-to-face learning experiences with online experiences” (p. 3) (Garrison & Kanuka, 2004). Since 2003, educational institutions have adopted different forms of online teaching combined with traditional face-to-face teaching, including hybrid course, blended course, and flipped classroom. In more detail, hybrid course focuses on replacing part of the traditional classroom with online learning (Riffell & Sibley, 2005). Blended course features some online elements, but less face-to face time than an equivalent traditional course (Mayadas et al., 2009). Flipped classroom often requires students to watch online videos prior to face-to-face lessons to increase engagement in interactive and higher-order activities, such as problem-solving, discussion, and debate (Kim et al., 2014) .

Blended learning is becoming popular topics in the academic community. More and more scholars have begun to focus on blended learning, such as curriculum hybrid learning reform (Chiu et al., 2012; Riffell & Sibley, 2005; Tekane et al., 2020), teacher factors (Georgouli et al., 2008; Lockee, 2021), and formative assessment (Baig et al., 2020; Gikandi et al., 2011). There have been several recent studies on blended learning, which described the current state and development Ashraf et al., 2021; Jiang et al., 2020; Kang & Kim, 2021; Kushairi & Ahmi, 2021; Lo & Hew, 2021). For example, Liu et al. (2016) conducted the meta-analysis on the effectiveness of blended learning for health professions learners based on 56 papers. For 56 papers from January 1990 to July 2019, Vallee et al. (2020) applied the meta-analysis to evaluate the effects of blended learning versus traditional learning in professional medical education. Leidl et al. (2020) analyzed 37 nursing research-related articles published between 2009 and 2019 and investigated the application of blended learning in nursing. Based on 103 papers related to community of inquiry published between 2008 and 2017, Stenbom (2018) revealed that the results provided by community theory are valid and reliable. According to the preferred reporting items for systematic reviews and meta-analyses guideline, Ashraf et al. (2021) explored 57 review articles associated with blended learning from 2012 to 2021, and studied the trends, gaps and future directions of blended learning. The aforementioned review either concentrated on a particular field of education (such as health or medical professional education) or conducted based upon meta-analyses rather than quantitative methodologies. These review studies had two limitations. First, as most studies used meta-analysis or hand coding, the number of articles was relatively limited (numbers ranged from 56 to 103). Second, the manual coding method involved a tedious and laborious coding process, and thus there might be inaccurate. Therefore, it is of great necessity to adopt a technical research method applicable to large-scale literature datasets to address the above limitations and outline the trends and directions of blended learning.

Topic modeling technology could provide a new method for large-scale literature research. As a natural language processing method, topic modeling could discover topics hidden in large amounts of textual data (Nielsen & Borjeson, 2019), which was considered to be more flexible and effective than alternative methods such as document clustering (Kuhn, 2018). It was demonstrated to be able to discover meaningful, valuable and appropriate topics in large amounts of text data (Jiang et al., 2016; Wu et al., 2021; Zhou et al., 2021). Recently, most of the methods have been applied in the social sciences (Isoaho et al., 2021; Kwok et al., 2021; Wu et al., 2021), with relatively few applications in blended learning.

Based on the above analysis, this study collects 3772 papers related to blended learning from 2003 to 2021 in the Web of Science core collection. Through the combination of topic modeling technology and word cloud analysis technology, all abstract texts are analyzed, aiming to draw the research topics and topic development trends of blended learning from a global perspective. In this study, there are two main research questions:

- Q1: What are the main research topics?
- Q2: What are the next development trends of various topics?

In response to the above two problems, the abstracts of 3772 paper documents were subjected to TF-IDF keyword extraction to form a keyword corpus. A time-phased word cloud based on the keyword corpus was built for word cloud feature evolution analysis. Additionally, LDA topic modeling technology created 7 well-defined topics on the corpus. The characteristics and development trends of each topic were analyzed. In this study, LDA topic modeling technology was applied to blended learning for the first time. Core parameters such as topic number were obtained through machine learning training. The data analyzed included 3772 documents in the Web of Science core collection from 2003 to 2021. Based on large-scale texts, the research topics and development trends of blended learning were excavated, thus providing theoretical and methodological references for related research on blended learning.

## Data and methods

**Fig. 1:**
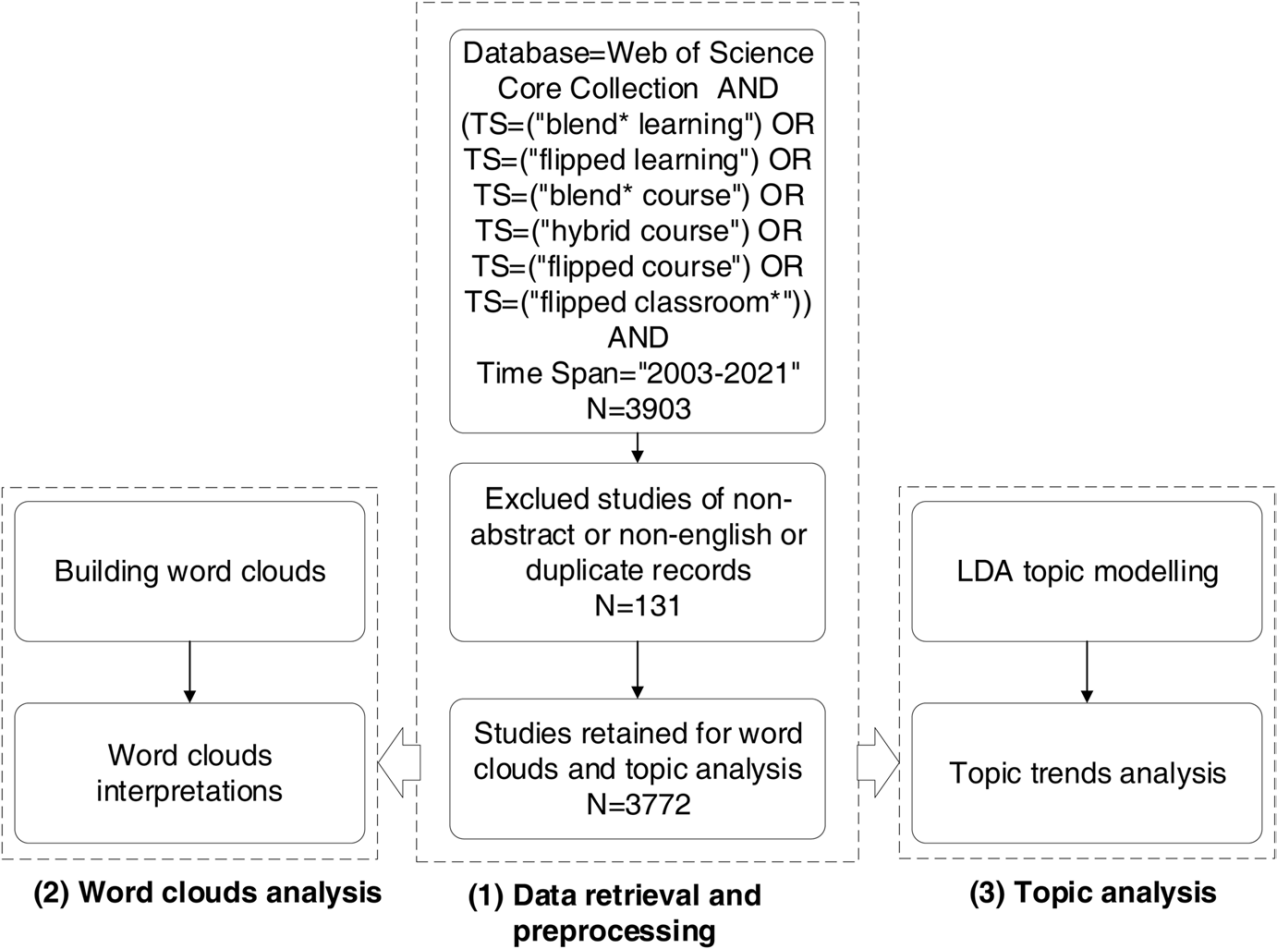
The flowchart of the dataset acquisition and analysis methodology

This study carried out quantitative analysis of plenty of articles related to blended learning, in which content analysis focused on the abstract content. The flowchart of the dataset acquisition and analysis methodology is shown in Fig. 1. The whole scheme was divided into three sub-processes, including data retrieval and preprocessing, word clouds analysis, and topic analysis.

### Data retrieval and preprocessing

**Fig. 2:**
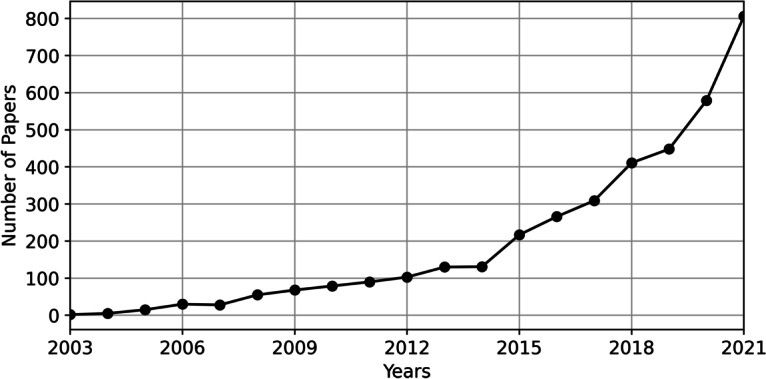
Annual scientific production per year (2003–2021)

The well-known literature retrieval platform Web of Science (WoS), which is the portal for all Social Sciences Citation Index (SSCI) and Science Citation Index (SCI) journals, was selected for this study. High-quality literature content could ensure high-quality research results. The query date was January 20, 2022, and thus that the data included all publications in the Web of Science database in 2021. The scope of literature search was limited to the literature with the paper type “Article” in the Web of Science Core Collection. A search string was formulated based on the relevant understanding and knowledge of blended learning, as well as the relevant search strings used in the study by Rasheed et al. (2020). The search string (TS= (“blend* learning”) OR TS= (“hybrid learning”) OR TS= (“flipped learning”) OR TS= (“blend* course”) OR TS= (“hybrid course”) OR TS= (“flipped course”) OR TS= (“flipped classroom”)) was keyed into the advanced search option of Web of Science database. TS refers to topic search, which includes searches for paper titles, abstracts, and keywords. Because the papers related to blended learning research appeared since 2003, we then specified the range of years from 2003 to 2021. All the relevant papers for answering our research questions were gathered. Initially, 3903 papers were retrieved. After checking the downloaded papers, 131 non-compliant papers were removed (including 98 papers without abstracts, 29 papers in non-English language, and 4 duplicate papers). In the end, 3772 papers were used for research. Paper information included title, abstract, year of publication, and journal title. As shown in Fig. 2, the download quantity of relevant literature presented an overall increasing trend.

According to Fig. 2, blended learning related research grew slowly until 2015, and then grew relatively rapidly in 2015–2018. With the COVID-19 outbreak in 2019, traditional teaching has been severely affected. On the contrary, blended learning courses have grown rapidly, which led to a linear upward trend in the number of related studies.

With abstract texts as the research object, this study carried out topic model mining. Before this, it was necessary to preprocess the data. Preprocessing included tokenize, removing punctuation, removing number, removing stop words, and lemmatize (Cho et al., 2017). Then, the initial word base was obtained. As for the interpretability of finally found topics, the initial word base was processed by the bi-gram algorithm (Bhagat & Pawar, 2018). Common collocation phrases were selected as corpus content for analysis, namely word1_word2 phrases. Similar phrases were added to the initial vocabulary. Ultimately, the initial corpus involved 407,096 terms in 3772 documents. To extract more representative keywords from the abstract, the initial corpus obtained above was processed through TF-IDF algorithm. Then, a keyword list with TF-IDF weights for each article was derived (Chowdhury, 2010). Among them, words with low weights were common words, such as “we” and “study”. Such words appeared in most articles, indicating that the content had weak representation. According to the method proposed by Hornik and Bettina (2011), keywords with weights lower than 0.1 were removed to obtain relatively great topic modeling results. After processing, a TF-IDF keyword corpus containing 3772 documents and 78,070 words was finally obtained.

### Word cloud analysis

Word cloud is a visual representation of a set of text documents that arranges and describes important words by font size, color, and space, which can help users quickly and efficiently understand the main content of a collection of documents (Chi et al., 2015). This analytical technique has been applied in many studies (Chen et al., 2020; Cho et al., 2017; Kwok et al., 2021). Considering the rapid changes in blended learning, the previously described TF-IDF keyword corpus containing 3772 documents and 78,070 words was set to a 4-year period (Cadirci & Gungor, 2021). WordCloud library of Python was used to generate the word cloud of each stage, in order to observe and understand the main research content in each stage.

### Topic modeling

Topic modeling is a probabilistic approach to understanding the hidden “semantic structure of document collections”, where the basic premise is that documents can represent multiple latent topics through the distribution of topic words (Blei et al., 2003). Over the past two decades, five topic modeling technologies have been introduced, namely latent semantic analysis (LSA), probabilistic latent semantic analysis (PLSA), latent dirichlet allocation (LDA), non-negative matrix factorization (NNMF) and Leximancer. With its accuracy, LDA is the best choice, which enables users to gain valuable insights and make data-driven decisions (Zhu et al., 2020).

In topic modeling technology, metrics usually include perplexity and coherence. As for perplexity, all words are assigned to an explicit topic as much as possible, and the modeling result is then measured by the likelihood value of the model. Modeling results often do not model human judgment well and are difficult to understand and interpret (Chang et al., 2009). The coherence is calculated based on the NPMI formula. Word pairs with high co-occurrence probability are assigned to a topic as much as possible. Therefore, the modeling results are highly understandable and interpretable. The NPMI formula is shown in formula (1) (Roeder et al., 2015).

1 $$\text{NPMI}\left({w}_{i},{w}_{j}\right)=\frac{\text{log}\frac{p\left({w}_{i},{w}_{j}\right)+\varepsilon }{p\left({w}_{i}\right).p\left({w}_{j}\right)}}{-\text{log}(p\left({w}_{i},{w}_{j}\right)+\varepsilon )}$$

p(w) is the probability that a word appears in a given document, and p(w_i_,w_j_) is the probability that these words appear together. NPMI measures the statistical dependence between word pairs, in which the higher the value, the better the model of the topic. This paper used the LdaModel module in the well-known natural language processing library Gensim in Python for topic modeling processing, and applied the coherence indicator NPMI to measure the model effect.

## Results

The display of analysis results was divided into three parts, including word cloud at each stage, topic modeling results, and topic trends analysis.

### Word cloud at each stage

**Fig. 3:**
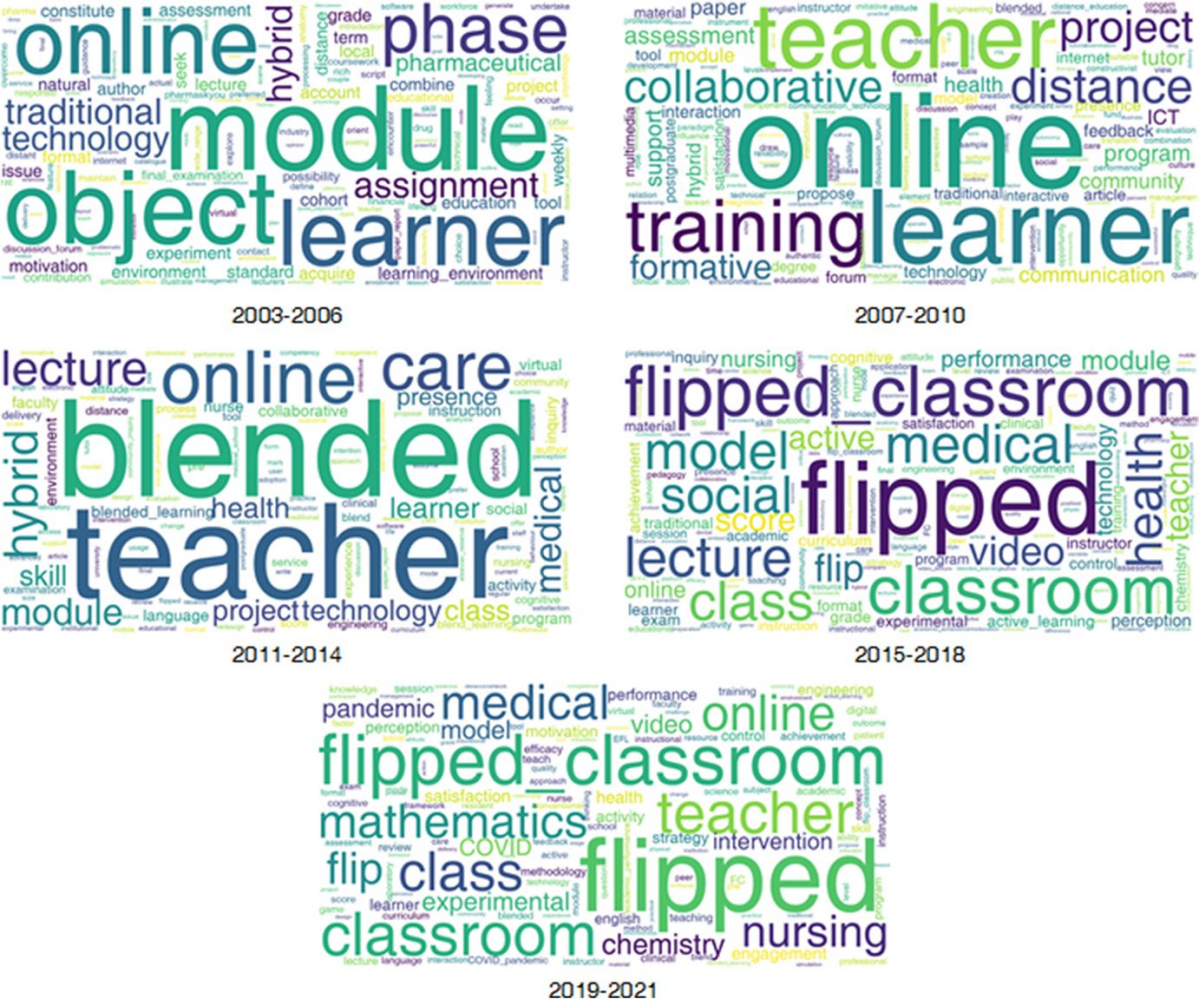
Most common words (2003–2021)

Word cloud can reflect the research feature words of the stage. Since blended learning changes rapidly, the phase period was set to 4 years (Cadirci & Gungor, 2021). According to different stages, the word cloud of each stage was generated. The results obtained are shown in Fig. 3.

During the period of 2003–2006, the prominent feature was hybrid course mode. At this stage, the learning object-related materials were managed online to support student learning (Alonso et al., 2005; Bender & Vredevoogd 2006; Ruiz et al., 2006). During the teaching process, some online learning modules were introduced, and part of the content was changed to online learning (Shaffer & Small, 2004). Teaching methods were also reformed, such as online assignments and interactive communication (Riffell & Sibley, 2005). By comparing new teaching methods and traditional teaching, the better hybrid course learning methods were sought, and the positive impact of technology on assessment and teaching was explored (Harrison, 2004; Ruiz et al., 2006).

During the period of 2007–2010, the prominent features were the study on online learning behavior of learners, such as the awareness and effect improvement of collaborative learning (Wang, 2010), interaction, communication, feedback, learning support and formative assessment (Crossouard & Pryor, 2009; Williams et al., 2008), job skills training based on blended learning (Lee, 2010), and teachers’ perceptions, attitudes and corresponding career development research on blended learning (Comas-Quinn, 2011; Owston et al., 2008).

During the period of 2011–2014, the research focus shifted from the online behavior in the previous stage to a blended course mode. Among them, the research on professional courses such as care, medical, and health was relatively prominent, for instance, the learning of medical knowledge and skills in the virtual patient module, and the use of network collaboration to examine learners’ performance and satisfaction (Guise et al., 2012; Kiviniemi, 2014; McLaughlin et al., 2014). In addition to increasing research on online community learning experience and presence (Shea & Bidjerano, 2012; Traver et al., 2014), teacher-related research was also a concern, such as teachers’ perspectives on new blended learning, instructional redesign, teaching experience, and teacher education (Bliuc et al., 2012; Jokinen & Mikkonen, 2013).

During the period of 2015–2018, blended course mode shifted to flipped classroom mode. Flipped classroom mode became a new model that scholars focused on, especially in the teaching of medicine and health. In these professional courses, students usually learned knowledge through videos and lectures in online platforms, and then discussed and expanded based on knowledge in class. This new teaching method could measure student performance, such as test scores, satisfaction, and skill improvement (Hew & Lo, 2018; Persky & McLaughlin 2017; Rathner & Schier, 2020). Among them, research on blended learning of nursing and chemistry courses occupied a certain proportion (Della Ratta, 2015; Seery, 2015). Compared with the traditional learning mode, the online learning environment provided by the flipped classroom mode allowed students to learn at their own time, with more obvious characteristics of repeated learning and active learning (Jensen et al., 2015).

During the period of 2019–2021, the research focus was still on flipped classroom mode. Compared with 2011–2014, teacher-related research in this period was more in-depth, such as teachers’ opinions, attitudes and information technology capabilities on flipped classroom, teacher training and careers with flipped classroom (Gonzalez-Gomez et al., 2019; Trujillo-Torres et al., 2020). In addition, research on flipped classroom for specialized courses in medicine and nursing still accounted for a large proportion. The proportion of research on flipped classroom in mathematics and chemistry courses increased (Bokosmaty et al., 2019; Hwang & Lai, 2017; Trujillo-Torres et al., 2020). The COVID-19 pandemic in 2019 has brought changes in various aspects, including student psychology, course engagement, performance, and online learning experiences during the pandemic Baloran, 2020; Bamoallem & Altarteer, 2022; Clark et al., 2021; Ibrahim et al., 2021; Yin & Yuan, 2021), blended learning methods have been adopted by teachers in response to the epidemic, and epidemic response strategies should be employed educational institutions (Ishimaru et al., 2021; Yang & Huang 2021). Some of these studies adopted experimental design method for comparing the learning effect of the intervention group with the control group (Zhang et al., 2020).

In general, the flipped classroom mode has been the focus of blended learning research for the past 7 years. In the early stage, the emphasis was on the hybrid course mode. In the middle stage, the blended course mode received attention. Since 2015, the flipped classroom mode has received a lot of attention so far.

### Topic modeling

#### Determination of optimal parameters

In topic modeling, the LDA model was considered to be the main research model with good results (Zhu et al., 2020). However, it should be noted that the LDA model was an unsupervised machine learning model. For the LdaModel module of the Gensim library in Python, the topic number K, the priori value of topic distribution α and the priori value of topic word distribution η involved in topic modeling needed to be predetermined. Furthermore, parameter determination could affect the final topic mining effect.

Metrics for the effectiveness of topic mining should be determined. Metrics are usually perplexity and coherence. Among them, coherence is mainly divided topics from the perspective of model interpretability. The higher the coherence, the better the interpretability of the model. The interpretability of the results is of great significance, which is related to the understanding of the results and the final application promotion. Therefore, coherence was chosen as the measure.

**Fig. 4:**
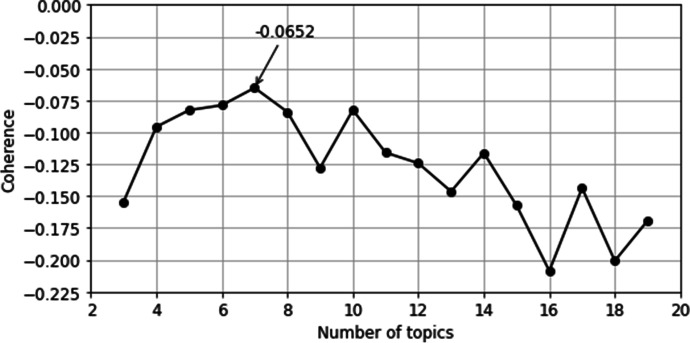
Topics number and coherence

Topic number K should be determined. The topic number determination test took 3–19 topics, and 1 step size. The priori value of topic distribution α and priori value of topic word distribution η were both set automatically. As for model simplification, the topic number was finally determined based on the coherence parameter. After testing, topic number and coherence effect values were obtained, as shown in Fig. 4. It could be seen from the figure that when the topic number was equal to 7, the coherence parameter value peaked at -0.0652. Therefore, topic number was set to 7, with automatically set values of α = 0.142 and η = 0.142.

**Fig. 5:**
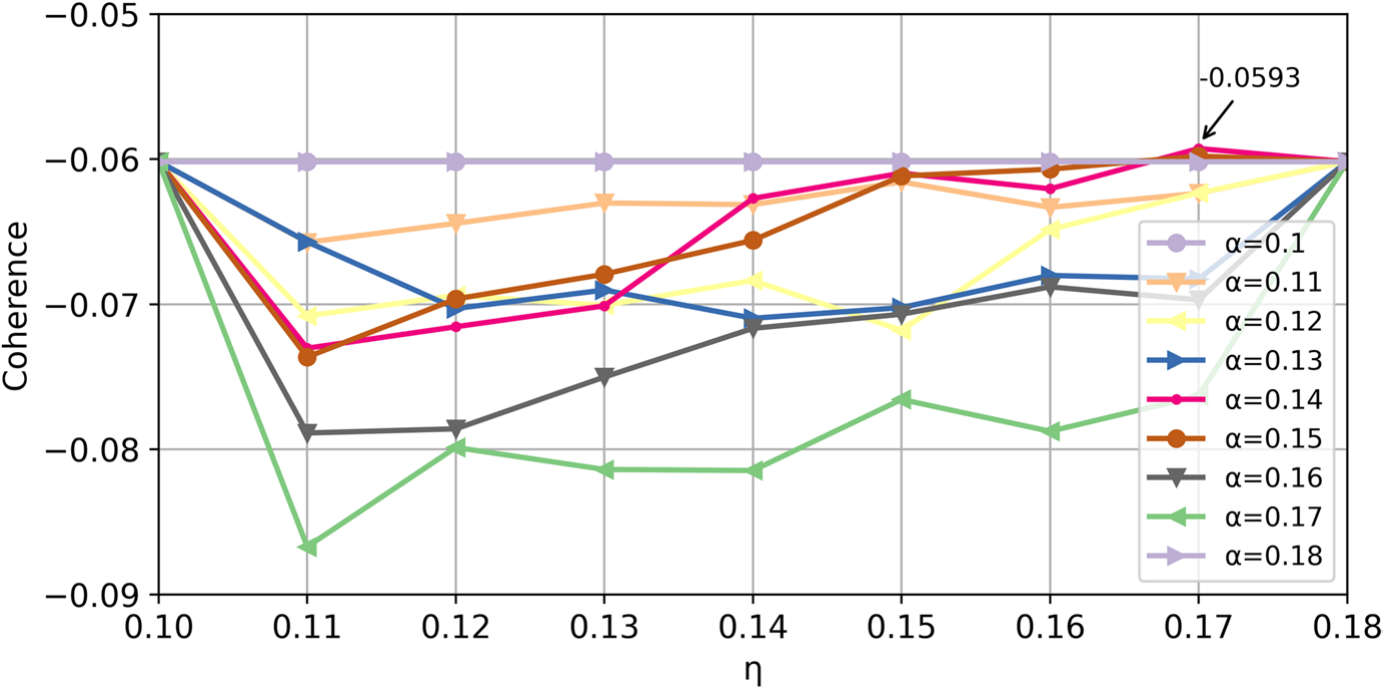
Coherence for different α and η

The optimal parameters α and η needed to be determined. These two parameters had a certain impact on the model results (Cho et al., 2017). After setting the topic number to 7, the auto-set value was adjusted in small ranges to further test the coherence parameter of the model and achieve the best results. By setting, α∈{0.10,0.11,0.12,0.13,0.14,0.15,0.16,0.17,0.18} and η∈{0.10,0.11,0.12,0.13,0.14,0.15,0.16,0.17,0.18}. Then, α and η were combined to form parameter pairs for topic modeling. The coherence score of the model was used to measure model performance. The result of the operation test was shown in Fig. 5. When α = 0.14 and η = 0.17, the coherence score reached a maximum of -0.0593, which was higher than the previous − 0.0652. Two experts reviewed the results of topic modeling. Ultimately, 7 topics proved to be clear and interpretable. Therefore, the optimal parameters α and η were determined, and the optimal model results for the 7 topics were obtained, as shown in Table 1.

#### Topic naming and topic details

After the model was determined, topic naming according to the results was done manually, which played an important role in the interpretability of the results. Traditional naming is mainly based on the probability p(w|t) of the topic words. Among them, keywords with high p(w|t) values are used for naming. In this way, the discrimination in topic naming is relatively low. In response to this, Sievert and Kenneth (2014) proposed a naming method that combines keywords with topic relevance. The correlation formula is as follows:

2 $$\gamma \left(w,t|\lambda \right)={\uplambda} \ \text{log}\left[p\left(w|t\right)\right]+\left(1-{\uplambda }\right)\text{log}\left[\frac{p\left(w|t\right)}{p\left(w\right)}\right]$$

where λ determines the weight given to the topic word *w* relative to its boost under topic *t*. In tuning λ, it was found that λ = 0.6 identified the most relevant and salient terms between topics. The resulting topics are shown in Table 1.

**Table 1:** Blended learning topics (2003–2021)

Category	Topic No.	Topic name	Representative terms	Proportion in the whole corpus
Learning elements	1	Learning outcomes	Satisfaction, achievement, active_learning, active, critical_thinking, literacy, methodology, traditional, traditional_ lecture	17.4%
	3	Teacher factors	Teacher, TPD(teacher professional development), technology, LMS(Learning Management Systems), reflective, philipsen, skill, adoption, pre_service	14.6%
	4	Presences	Presence, social_presence, CoI(Community of Inquiry), cognitive_presence, social, community, metacognitive, relationship, perception	14%
	6	Formative assessment	formative_assessment, assessment, online, SRL(self-regulated learning), module, laboratory, curriculum, exam, behavior	13.6%
Courses education	2	Chemistry & mathematics education	Chemistry, organic, FC(flipped classroom), MOOC, TBL(team-based learning), game, mathematics, safety, podcasts	15.5%
	5	Nursing & medical education	Nursing, clinical, medical, CBT(cognitive-behavioral therapy), pathology, antibiotic, surgical, physiology, psychiatry	13.9%
	7	Health education	Health, video, AR, disaster, public_health, global_health, pediatric, wheelchair, PBL(project-based learning)	11%

#### Topic visualization

**Fig. 6:**
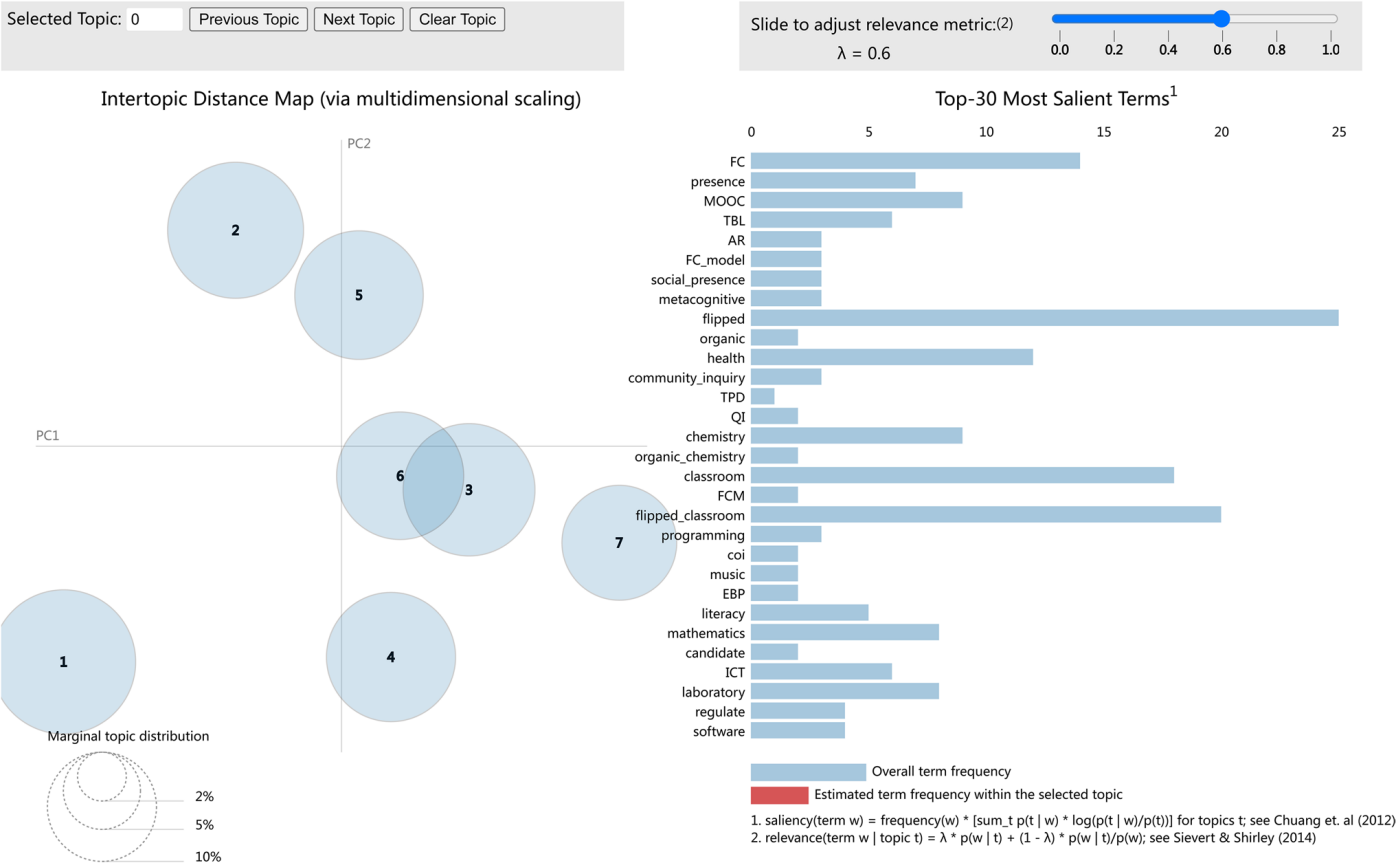
The visualization of the global topic model. URL: http://research.lechouchou.club/model/topics_vis.html#topic=0&lambda=0.6&term=

Topic visualization used pyLDAvis software. Figure 6 presents the results of topic model as a whole. Through a multi-dimensionally zoomed model view, the pyLDAvis visualization method presents information about the meaning, popularity, and relationships of each topic. The left panel presents the distance between topics, where the size of the circle indicates popularity. With horizontal bar graph, the right panel lists the most representative terms in each topic, along with detailed structural information. Graphically, the topics were well differentiated with well-balanced popularity. We uploaded the entire model to Internet and made it accessible via a dynamic web page. Readers can explore the topic model for their own particular interests using an interactive, intuitive interface, as displayed in Fig. 6.

### Topic change over time and topic trends

#### Analysis of topic trends

**Fig. 7:**
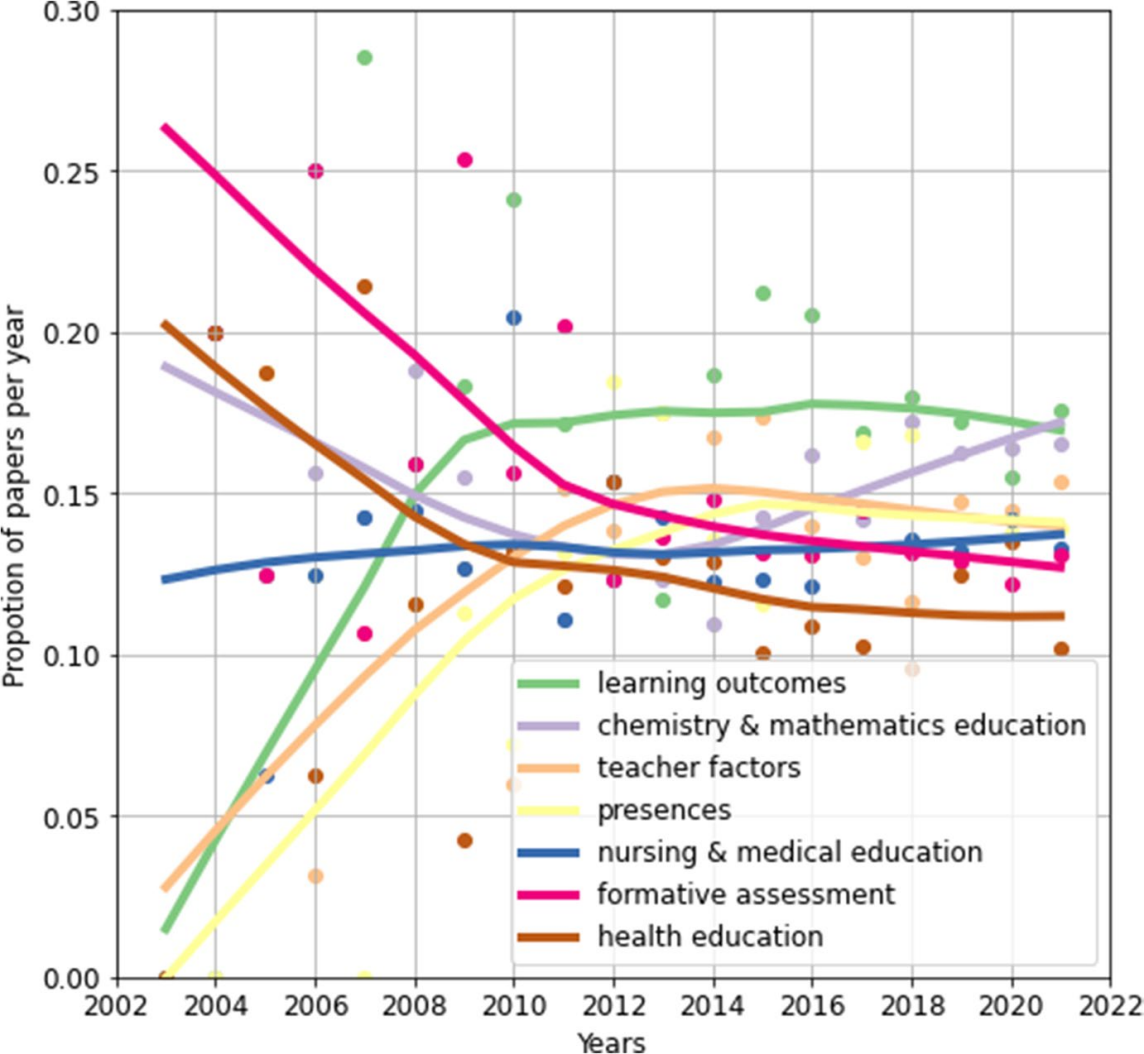
Trends of the topics

Trends of topic changes over time, which can provide researchers with valuable content. The annual proportion of the number of papers on each topic reflects the degree of attention to this topic. The percentage data changes every year, and some changes fluctuate wildly in the short term. A scatterplot of the topic’s annual share data can reflect this change. In order to obtain a relatively stable change trend of the topic, based on the annual proportion data, reference is made to the literature (Cho et al., 2017), and the Loess algorithm is adopted to smooth the annual proportion data to obtain the smoothed data. The smoothed data is used to draw a smooth curve, which can reflect the relatively stable changing trend of the topic. The results are shown in Fig. 7.

In the early stages, the topic “formative assessment” was the most popular, but with overall decline (13.6%). Notable features of the topic included formative assessment, assessment, online, self-regulated learning, and exam. For example, Baig et al. (2020) utilized the Blackboard (Bb) system for formative online assessment and found that online assessment improved students’ final exam performance. Broadbent et al. (2021) investigated the impact of self-regulated learning on formal assessment by comparing online and blended learners. Before 2010, the topic “formative assessment” was the most popular. When this topic was introduced into blended learning in e-learning systems, researchers paid extensive attention to it, since assessment was the core of formal higher education (Gikandi et al., 2011). Nevertheless with the diversification of research, the proportion of this topic was obviously declining, and the proportion of this topic in 2021 slipped to the bottom second.

“Health education” topic trends similarly to the “formative assessment” topic (11%). Under this topic, the research focus was mainly on professional courses in health education. For example, Chiu et al. (2012) used a blended learning approach to train public health nursing students in Ohio in a “disaster surge capability course”. For the “anatomy” flipped classroom of health science students, (Ferrer-Torregrosa et al., 2016) conducted a comparative study of three different teaching tools, augmented reality, video and note. Stallwood et al. (2020) experimented with teaching reform based on a blended learning approach in a “global health course” for 25 graduate students in different health professions and public health fields. From 2003 to 2006, this topic ranked second in the research literature, after the topic “formative assessment”. With the diversification of research, the enthusiasm of scholars gradually weakened, and the proportion of this topic dropped to the last in 2021.

The topic “chemistry & mathematics education” presented a concave development curve (15.5%). The subject included blended learning research in chemistry and mathematics courses. For example, Piotto et al. (2003) introduced an Internet-based scientific visualization teaching system to promote the understanding of chemical principles. For a second-year organic chemistry curriculum in South Africa, Tekane et al. (2020) explored the perceptions and preferences of blended learning for student learning support. Hwang and Lai (2017) proposed an interactive e-book approach to support flipped learning in math classes. This topic accounted for a relatively high proportion in the early stage, especially for chemistry course research, but it entered a low point in 2012. Research on both classes rose rapidly with the addition of math courses, which jumped to No.1 in 2021.

The topic “learning outcomes” had the fastest rise in popularity (17.4%), which focused on student outcomes after blended learning. Among them, the obvious feature words included satisfaction, academic performance, active learning, and critical thinking. For example, Peterson (2016) compared flipped classroom with traditional teaching in a “statistics class” and found that students who participated in flipped classroom had higher grades and satisfaction. Mortensen and Nicholson (2015) found that flipped classroom was an active learning experience for students and could improve students’ critical thinking. By applying the combination of blended learning and BOPPPS mode (BL-BOPPPS) to the introductory health service management (HSM) course for Chinese health management majors, Ma et al. (2021) found that the combined mode could improve students’ performance and satisfaction. In 2003, the proportion of research on this topic was less than 0.05. However, this topic gained the attention of scholars and the number of studies has increased rapidly. In 2010, the thematic trend line peaked and then remained at the top until 2021.

The trend line for the topic “teacher factors” was similar to that for the topic “learning outcomes”, with lower popularity (14.6%). This topic involved teacher professional development, the use of learning management systems, and teaching-related technologies and skills in blended learning. For example, Georgouli et al. (2008) introduced LMS (Learning Management Systems) into traditional lectures to effectively support student learning. Lockee (2021) explored the use of blended learning for professional competency development training for teachers during the COVID-19 pandemic. In 2003, the proportion of research on this topic was less than 0.05. After that, the proportion of research gradually climbed and reached No. 2 by 2013, which dropped slightly to No. 3 by 2021.

The “presenses” topic trend line was bowed upwards (14%). With community of inquiry theory as framework, this topic studied presences in online learning, including social presence, cognitive presence, and teaching presence (Garrison et al., 2000). For example, Garrison et al. (2010) explored the causal relationship between three kinds of presences in the CoI framework. Ozturk (2015) investigated the high correlation between social presence, cognitive presence, and teaching presence in Facebook’s learning community, and proposed that Facebook is a suitable online environment for the CoI framework. Law et al. (2019) explored the mediating effect of CoI among students’ course selection, motivation and learning performance. In 2003, the proportion of research on this topic was 0. After that, the proportion of this topic gradually increased to a peak in 2015, and then decreased slightly in 2021.

The trend line for the “nursing & medical education” topic was flat horizontal (13.9%). This topic included research in blended learning for nursing and medical education majors. For example, Chen et al. (2009) studied the self-regulation strategies of Taiwanese registered nurses in blended curriculum. Parker et al. (2020) employed multiple strategies and techniques for targeted anatomical pathology training. The proportion of this topic has maintained a horizontal trend from 2003 to 2021. In other words, this particular topic continues to receive attention from researchers.

In general, thematic trends can be divided into two stages. Before 2013, the topics explored the implementation of blended learning in professional courses education and formative assessment based on learning systems. After 2013, the focus shifted to learning outcomes, teacher-related factors, and presences in the blended learning process. It indicates that the research focus has shifted from the previous courses education topics to the recent blended learning key element topics, which reflects the depth and breadth of concerns.

#### Hot and cold topics

On the basis of trend analysis, hot and cold topics were analyzed. Differently, hot and cold topics were elaborated in a statistical sense.

**Fig. 8:**
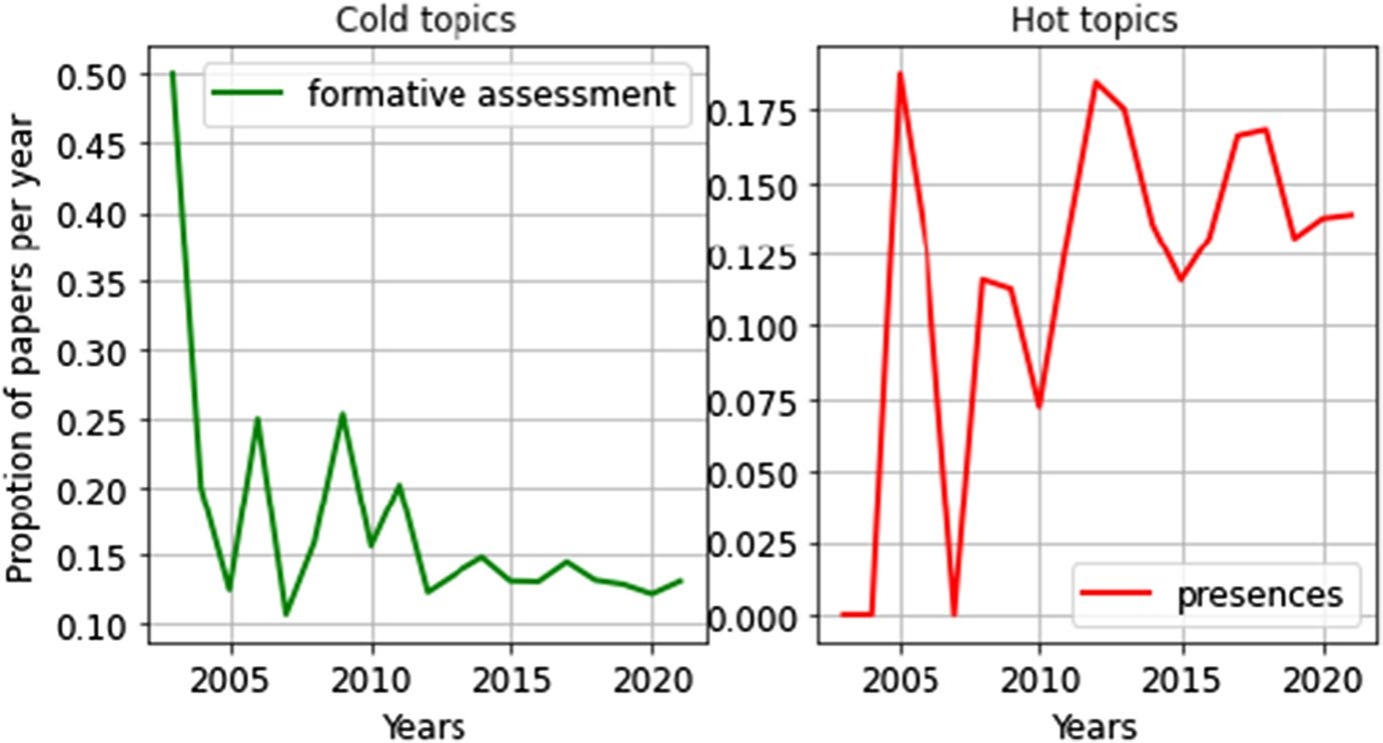
Hot and cold topics in blended learning (2003–2021)

There is a strong trend in the field of study, namely drastic increase or significant decrease. According to Griffiths and Steyvers (2004), topics with significant increase or decrease in popularity between 2003 and 2021 were attempted to be defined. Table 2; Fig. 8 summarize the analysis results.

**Table 2:** Statistically significant topic trends (2003 ~ 2021)

Trend	Topics	*p* value
Negative trend	Formative assessment	*
Positive trend	Presences	*
No trend	Learning outcomes, Chemistry & mathematics education, Teacher factors, Nursing & medical education, Health education	NS

*Notes*: *p < 0.05, NS = non-significant

To understand the hot and cold trends of different topics, the linear trends of the proportion of topic papers per year were analyzed (as shown in Table 2). When the significance was 0.05, the proportion of the topic “formative assessment” had an obvious downward trend, with statistical significance of 0.0173. The proportion of the topic “presences” increased significantly, with statistical significance of 0.0456. Figure 8 shows the detailed curve of the proportion of papers with hot and cold topics. The rising and falling trends for the other 5 themes are not statistically significant.

The trending topic graph is the most attractive topic indicator, which depicts the popularity of trending topics. Over the past 19 years, the community of inquiry theory proposed by Garrison et al. (2000) has been a popular theoretical analysis framework in blended learning research. Many scholars have studied the relationship between presences and learning effect in courses teaching reform based on this theoretical framework. In addition, some scholars have studied the relationship between the three presences and their influence on the learning effect mode. According to the results of popular topics, the topic of “presences” received more attention.

Additionally, unpopular topic maps relate to topics that people are losing interest in. Although educational evaluation is the core content of education, when the online learning system introduced blended learning, many scholars used the electronic learning records in the learning system to conduct formative assessment research on various learning strategies and behaviors. Over time, the proportion of such assessment studies has decreased significantly. According to the results of the less popular topics, the topic of “formative assessment” received significantly less attention.

The attention of the other five topics did not change significantly, but with considerable position. According to Fig. 7, the topic “learning outcomes” had not increased statistically significantly in recent years, but with the highest proportion. This showed that scholars were still concerned about this topic. The four topics “Chemistry & mathematics education”, “Teacher factors”, “Nursing & medical courses”, and “Health courses” were similar to the topic “Learning outcomes”, which occupied a considerable proportion.

## Discussion

Through word cloud analysis and topic modeling of 3772 paper abstracts in the Web of Science core collection, word cloud at each stage, 7 well-defined topics and their development trends were obtained. Based on the research results, the research findings involved five aspects, which were shift of topic focus, characteristics of courses education topic, characteristics of learning elements topic, hot and cold topics, and key modes of blended learning.

In terms of shift of topic focus, the proportion of courses education topics gradually decreased. Conversely, the topics of learning elements grew rapidly and then dominated for a long time. From 2003 to 2010, the e-learning system was gradually introduced into students’ learning, and researchers began to explore the transformation of traditional learning into blended learning from specific courses, such as health, nursing and medicine, chemistry. Blended learning reforms were carried out, and blended learning research in these course topics accounted for most of the papers at this stage. However, with the progress of the research, scholars quickly developed a strong interest in the study of key elements of blended learning, and related papers on students’ learning outcomes, teacher-related factors, and presences in the learning process increased rapidly. After 2010, the proportion of research institutes in these three topics significantly exceeded the research on course topics, and then in a dominant position for a long time between 2010 and 2021. This is the first and most profound finding of this study.

Courses education topics focused on a few curriculum categories. Currently, blended learning studies involved health, nursing, and medical courses, which were consistent with that of Ashraf et al. (2021). With the rapid development of the Internet, medical knowledge on the Internet has increased significantly. The use of the Internet was necessary and important in healthcare (Masic, 2008). Because the relevant teachers and students have information technology literacy earlier, they reform and explore blended learning earlier than other types of courses. Moreover, chemistry and mathematics courses were more abstract (Hwang & Lai, 2017; Piotto et al., 2003), such as organic chemistry in chemistry courses and calculus in mathematics courses. In order to achieve better learning effects, teachers combined learning resources on the Internet with traditional face-to-face classrooms. Reforms were attempted while taking advantage of both approaches. Blended learning research in these five categories dominated. Relatively speaking, there was a lack of research on other types of courses.

Learning elements topics focused on both students and teachers. There was relatively little research on educational institutions. In blended learning, satisfaction, academic performance and critical thinking were important manifestations of student learning outcomes. These contents were concentrated in the mainstream topic “learning outcomes”, accounting for the highest proportion. For the reform of blended learning, teachers’ further professional development, technical ability, teaching reflection and teaching skills were all important influencing factors, which were mainly reflected in the theme of “teacher factors”. In addition to students and teachers, blended learning also involved educational institutions. The attitudes and measures of educational institutions towards blended learning have an impact on the reform of blended learning. However, the amount of such literature is insufficient. This was consistent with the findings of Ashraf et al. (2021).

Two topics of “presences” and “formative assessment” need special attention. This study found that “presences” was a trending topic with significantly increased attention, which should receive more attention in follow-up research. “Formative assessment” is an unpopular topic with significantly reduced attention, which is supported by Jiang et al. (2020). Scholars should study this topic carefully. The attention of the other 5 topics does not change significantly but accounts for the same proportion, which should receive continuous attention.

The flipped classroom mode has become the focus of blended learning research in recent years. Compared with the early hybrid course mode and the mid-term blended course mode, this mode requires students to learn basic knowledge through the Internet before face-to-face class, and extend and expand the understanding and application of basic knowledge in the classroom. The advantages of this mode are to improve learning outcomes, enhance learning flexibility, and increase teacher-student interaction (Akcayir & Akcayir, 2018), which is beneficial to students’ in-depth mastery of relevant knowledge, enhancement of application ability, and improvement of critical thinking. Scholars can focus on the research of this mode.

## Conclusions

This paper aims to draw the topic context and development trend of research on blended learning from a global perspective. Through word cloud and topic modeling technology, 3772 papers in the Web of Science core collection were analyzed. The distribution changes of word cloud at each stage and 7 well-defined topics and their development trends were obtained. Then, the overall research context of blended learning was drawn. Among them, the profound discovery lies in the shift of the focus of topic development trend. In more detail, the overall research proportion of courses education topics gradually decreased, while the proportion of learning elements topics of blended learning increased rapidly and dominated for a long time. Courses education topics focuses on health, nursing, medicine, chemistry and mathematics. Learning elements topics focus on students and teachers, and less on educational institutions. The “presences” topic is a popular topic with significant increase in interest, while the “formative assessment” topic is a less popular topic with significant decrease in interest. The attention of the other five topics does not change significantly but accounts for a similar proportion. Compared with hybrid course mode and blended course mode, flipped classroom mode has become the focus of blended learning research in the past seven years. This study contributes to both theoretical results and methodological innovations. In this study, LDA topic modeling technology was applied to blended learning for the first time. Core parameters such as topic number were obtained through machine learning training. The data analyzed included 3772 documents in the Web of Science core set from 2003 to 2021. The research topics and development trends of blended learning are excavated based on large-scale texts analysis for the first time, thus providing theoretical and methodological references for related research on blended learning.

Despite some contributions, this study suffers from two limitations. First, the dataset used affects the generalizability of the findings. In more detail, the literature in the Web of Science core library is in full English, which may lead to limitations in the general applicability of the results. Second, the main analysis is the abstract of the paper, rather than the full text. Therefore, there is a limit to the granularity of results. Future research should consider exploring the full text of authoritative articles with multiple language versions.

## Data Availability

The datasets generated during and/or analysed during the current study are available from the corresponding author on reasonable request.
